# Renoprotective Effect of Danhong Injection on Streptozotocin-Induced Diabetic Rats through a Peroxisome Proliferator-Activated Receptor *γ* Mediated Pathway

**DOI:** 10.1155/2018/3450141

**Published:** 2018-04-11

**Authors:** Xue Yang, Xiang Xiao, Hailian Wang, Yi Li, Li Wang, Guisen Li, Shaoping Deng

**Affiliations:** ^1^Renal Division and Institute of Nephrology, Sichuan Academy of Medical Science and Sichuan Provincial People's Hospital, School of Medicine, University of Electronic Science and Technology of China, Chengdu 610072, China; ^2^Southwest Medical University, Luzhou 646000, China; ^3^Institute of Organ Transplantation, Sichuan Academy of Medical Science and Sichuan Provincial People's Hospital, Chengdu 610072, China

## Abstract

The aim of the study was to investigate the protective effect of Danhong injection (DHI) on diabetic kidney disease and explore the potential mechanisms. Diabetic kidney disease was induced by unilateral nephrectomy, high-fat diet, and streptozotocin. After DHI administration, the renal function deterioration, 24-hour total urine protein excretion, and elevated serum lipid levels were reversed to some extent, and the renal pathological damage was also ameliorated. The KEGG pathway enrichment analysis demonstrated that the PPAR*γ* signal pathway was significantly upregulated in DH group. And the increased expressions of PPAR*γ* and UCP-1 were confirmed by immunohistochemistry, whereas the p38MAPK was significantly decreased. These data show that DHI could delay the progress of DKD, and the effect might be achieved in part by activating the PPAR*γ* signaling pathway.

## 1. Introduction

Diabetic kidney disease (DKD) is one of the diabetic microvascular complications characterized by proteinuria and progressively deterioration of renal function. Until today, a variety of treatment strategies still could not completely prevent the progress of DKD into end-stage renal disease (ESRD). Over the past decades, scientists have been trying to find different ways and agents to treat DKD, but the results were not satisfactory [1–3].

For example, recently, a multinational prospective randomized controlled trial involving 8561 patients with type 2 diabetes mellitus and chronic kidney disease was prematurely terminated because of the severe adverse events of aliskiren, including increased risk of acute kidney injury, hyperkalemia, hypotension, and stroke [4]. Another study indicated that, among patients with type 2 diabetes mellitus and stage 4 chronic kidney disease, bardoxolone methyl, a nuclear factor E2 related factor-2 activator, did not reduce the risk of ESRD or death from cardiovascular [5]. Moreover, DKD was still the leading cause of ESRD in the United States [6]. A recent report revealed that chronic kidney disease related to diabetes has become more common than chronic kidney disease related to glomerulonephritis in China [7]. Therefore, more effective drugs that can delay or prevent the progress of DKD are necessary.

Danhong injection (DHI) was extracted from Salvia miltiorrhiza and Carthami tinctorii and used widely for the treatment of cardiovascular diseases in China. Qualitative and quantitative characterization of DHI was effectively evaluated by high-performance liquid chromatography with diode array detection (HPLC-DAD) [8, 9]. Previous studies indicated that DHI could decrease the levels of urinary albumin excretion rate (UAER) and 24-hour urinary protein in patients with DKD [10, 11]. In experimental DKD rats, renal hypertrophy and 24 h urinary protein excretion were ameliorated by Tanshinone IIA, an active ingredient of Salvia miltiorrhiza [12]. Liu et al. reported that DHI inhibited the development of diabetic retinopathy and nephropathy in diabetic db/db mice [13]. These data suggested that DHI could effectively delay the progress of DKD, but the molecular mechanisms for the renoprotective effects of DHI were still unclear. In this study, we established a DKD rat model to explore the renoprotective effect of DHI and investigate the underlying mechanisms.

## 2. Materials and Methods

### 2.1. Reagents

Rabbit anti-PPAR*γ* and anti-UCP-1 polyclonal antibodies were purchased from Proteintech Group (Chicago, IL, USA). Rabbit anti-P38MAPK polyclonal antibody was obtained from Cell Signaling Technologies (Danvers, MA, USA). Streptozotocin (STZ) was purchased from Sigma-Aldrich (St. Louis, MO, USA).

### 2.2. Quality Control of DHI

Danhong injection was purchased from Heze Buchang Pharmaceutical Co., Ltd. (Shandong, China) (drug approval number: Z20026866). According to the criteria of China Food and Drug Administration, the quality control standard of DHI was that the total amounts of danshensu (molecular formula: C_9_H_10_O_5_) and protocatechuic aldehyde (molecular formula: C_7_H_6_O_3_) should not be lower than 0.5 mg in 1 mL injection analyzed by high-performance liquid chromatography (HPLC). Meanwhile, the total flavonoids detected by visible spectrophotometry should not be lower than 5.0 mg/mL against rutin (molecular formula: C_27_H_30_O_16_) [14].

### 2.3. Animals

Male Sprague-Dawley rats (4 to 5 weeks) weighing 200–250 g were obtained from Dashuo Biotechnology Co., Ltd. (Chengdu, China). Rats were kept in a room with 12 h light-dark cycle (temperature 18–29°C and relative humidity 40–70%) and were free to get food and drink. We tried our best to reduce the number of animals and suffering during the experiments. The protocols for in vivo study with rats accorded with the Guide for the Care and Use of Laboratory Animals published by NIH, and all processes conformed to international guidelines on the ethical use of animals. This study was approved by Institutional Review Boards of the Sichuan Academy of Medical Sciences and Sichuan Provincial People's Hospital.

### 2.4. Animal Model

After a week of adaptive feeding, all animals were given unilateral nephrectomy and then fed with a high-fat diet for 4 weeks. Twelve rats were selected as the control group (NM group, *n* = 12), and the remaining rats were fasted for 12 hours and then injected intraperitoneally with STZ (38 mg/kg). After 72 hours and 7 days, the blood was withdrawn from the tail vein to measure glucose levels. If the blood glucose levels in NM group were normal and in model group were more than 16.7 mmol/L^−1^, it is recognized as a successful model. Forty rats with diabetic kidney disease were randomly divided into two groups: DHI injected group (DH group, *n* = 20) and saline injected group (NS group, *n* = 20). DH group was given Danhong injection intraperitoneally daily (2 ml/kg) for two weeks; NM group and NS group were given saline intraperitoneally daily (2 ml/kg) for two weeks.

### 2.5. Determination of Renal Functions and Serum Lipid Levels

Four rats were randomly chosen in each group to collect 24 h urine in metabolic cages at the 2nd, 6th, and 10th week after drug administration. The urine samples were used to detect 24-hour urine total protein (24 h TP). After anesthetizing the selected rats, abdominal aorta blood samples were gathered to determine blood urea nitrogen (BUN), serum creatinine (Cr), cystatin C (Cys-C), total cholesterol (TC), triglyceride (TG), low-density lipoprotein cholesterol (LDL-C), and high-density lipoprotein cholesterol (HDL-C) by automatic biochemical analyzer (Hitachi, Japan).

### 2.6. Identification of DEGs and KEGG Pathway by Enrichment Analysis

Total RNA from the kidneys in the three groups (*n* = 3 per group for each time point) were isolated by TRIzol® Reagent (Invitrogen Life Technologies), and then RNA was qualified and quantified by NanoDrop ND-1000. Samples were marked by Arraystar RNA Flash Labeling Kit and hybridization was performed by Agilent SureHyb. The hybridized arrays were washed, fixed, and scanned by using the Agilent DNA Microarray Scanner (part number G2505C). Agilent Feature Extraction software (v11.0.1.1) was used to capture the chip probe signal values. Agilent GeneSpring GX v12.1 software was used to standardize chip and select the differential expression mRNA (fold change *⩾* 2.0, *P* value ⩽ 0.05). Then the differentially expressed mRNA was analyzed based on Kyoto Encyclopedia of Gene and Genomes (KEGG) pathway.

### 2.7. Determination of Morphology and the Expression of PPAR*γ*, UCP-1, and p38MAPK in the Kidney

After the rats were sacrificed at different time points mentioned above, the right kidneys were removed and fixed in 4% paraformaldehyde followed by embedding in paraffin. To evaluate the renal pathological changes and the glomerular volume, the kidney 5 *μ*m cross paraffin sections were prepared and stained with periodic acid-Schiff (PAS). The slides were also used to detect the expression of PPAR*γ*, uncoupling protein-1 (UCP-1), and p38MAPK by immunohistochemistry staining. Three sections were stained from each kidney and there were 4 rats in each group.

### 2.8. Statistical Analysis

Data were presented as mean ± SD and the significant difference was considered at *P* < 0.05. When the variance is homogeneous, use one-way ANOVA; if not, use rank sum test. Statistical analysis was performed using SPSS software (version 16.0; SPSS Inc., Chicago, IL).

## 3. Results

### 3.1. Effects of DHI on Renal Function

**Figure 1:**
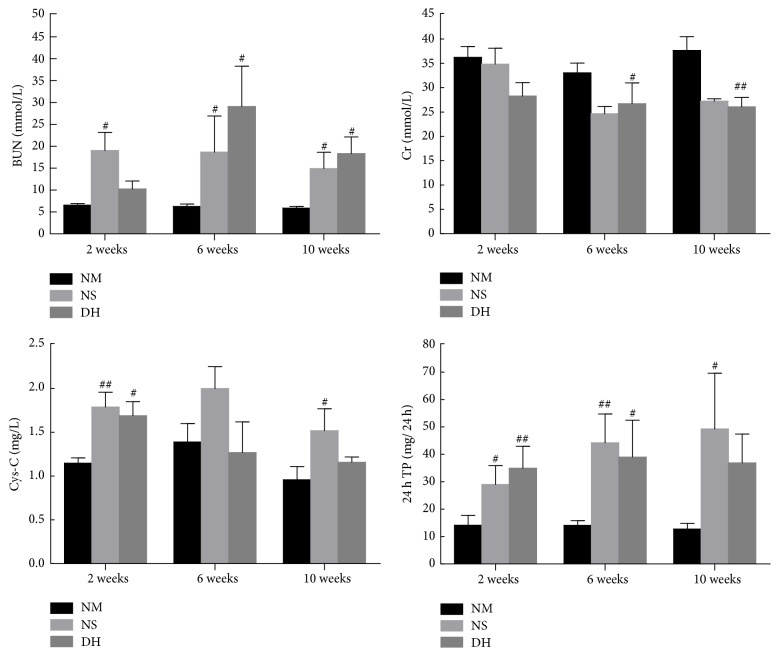
Effects of DHI on renal function and 24-hour urine total protein. The levels of BUN, Cr, Cys-C, and 24 h-TP were measured at the 2nd, 6th, and 10th week after administration by automatic biochemical analyzer. ^#^*P* < 0.05 and ^##^*P* < 0.01 versus the control group (NM).

We examined the serum levels of BUN, Cr, Cys-C, and 24 h-TP to determine the therapeutic effect of DHI for DKD rats. The levels of BUN, Cys-C, and 24 h TP in the NS group were significantly higher than those in the NM group. After DHI intraperitoneal administration for 14 days, the changes of serum BUN and Cr showed a downward trend in DH group compared with NS group (Figure 1). Moreover, although the 24 h TP levels of rats in DH group were higher than NS group at the end of the 2nd week, Danhong injection decreased 24 h TP levels at the 6th and 10th week. In addition, the Cys-C levels of rats in the DH group were lower than that in the NS group at each time point after DHI administration.

### 3.2. Effects of DHI on Kidney Morphology

**Figure 2:**
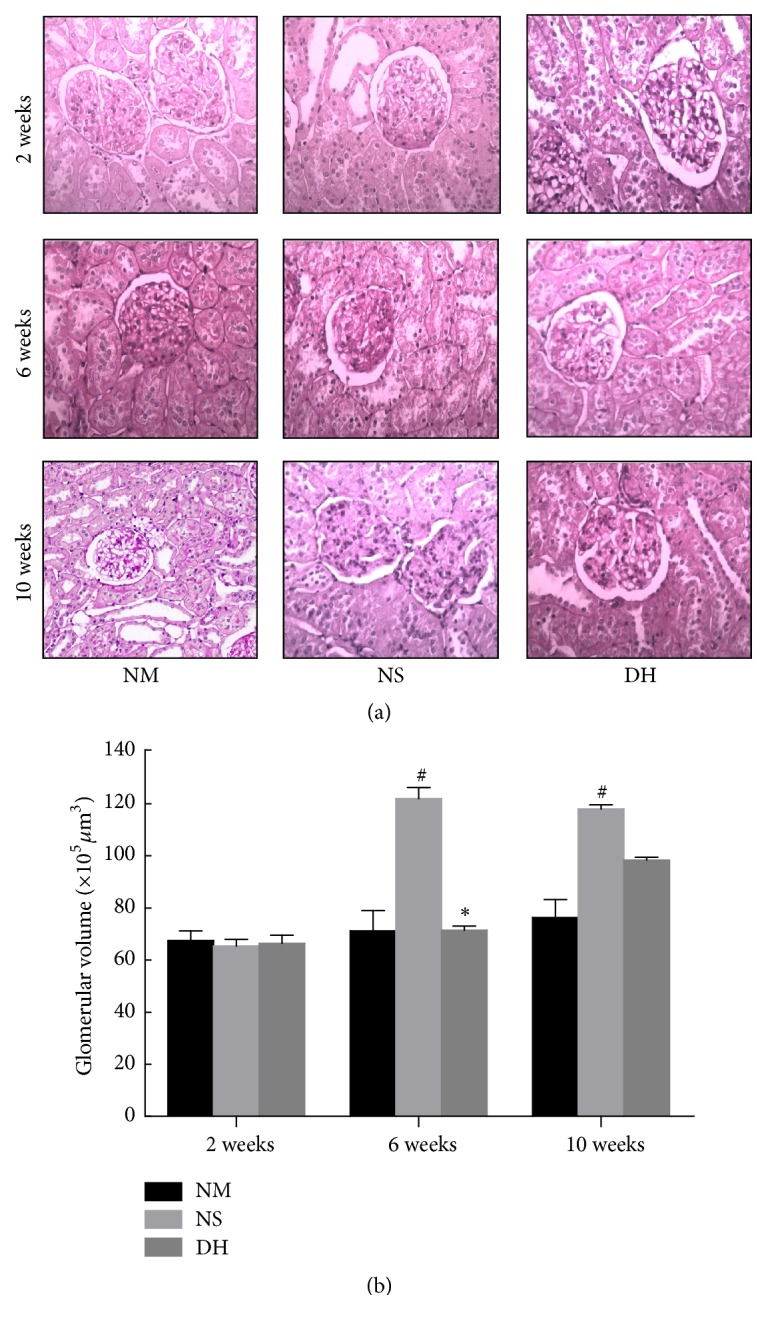
Effects of DHI on renal morphology and glomerular volume. After the rats were sacrificed at the 2nd, 6th, and 10th week, the kidney 5 *μ*m cross paraffin sections were prepared and stained with PAS (a) (scale bar: 500 *μ*m), and the glomerular volumes were quantified (b). ^#^*P* < 0.05 versus the control group (NM); ^*∗*^*P* < 0.05 versus the saline injection group (NS).

The characteristic pathological changes in DH group and NS group included increased glomerular volume, mesangial matrix proliferation, increased mesangial cells, and thickening basal membrane. Comparing with NS group, the DH group showed alleviated pathological damage at each time point after DHI administration (Figure 2(a)). As showed in Figure 2(b), there was no significant difference about the glomerular volume among each group at the 2nd week. But at the 6th and 10th week, glomerular volume in the DH group was smaller than that of the NS group, especially at the 6th week (*P* < 0.05).

### 3.3. Effects of DHI on Serum Lipid Levels

**Figure 3:**
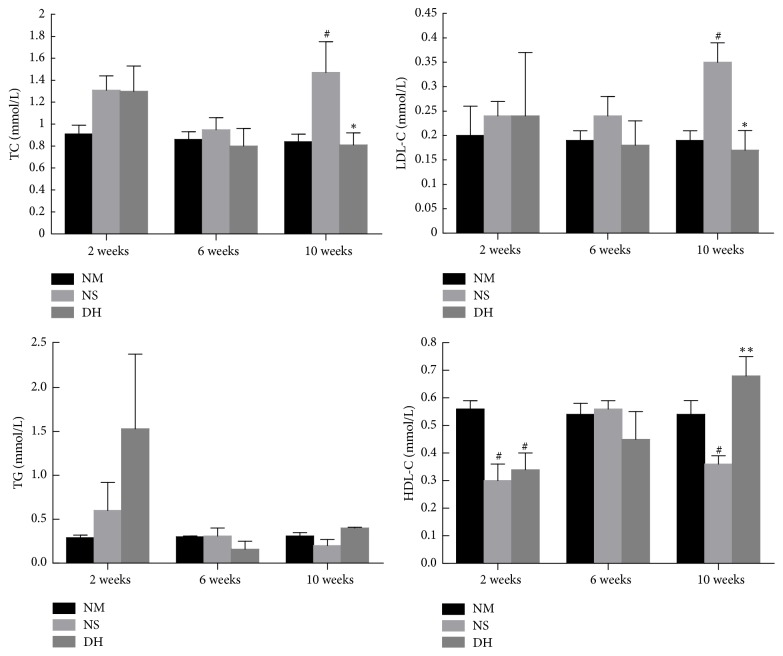
Effects of DHI on serum lipid levels. To test the circulating lipid profiles, the blood samples were collected at the 2nd, 6th, and 10th week after administration. ^#^*P* < 0.05 versus the control group (NM); ^*∗*^*P* < 0.05 and ^*∗∗*^*P* < 0.01 versus the saline injection group (NS).

To assess the circulating lipid profiles, the serum samples were collected to detect the levels of TC, TG, LDL-C, and HDL-C. The results indicated that DHI significantly reduced TC and LDL-C levels (*P* < 0.05) and elevated HDL-C (*P* < 0.01) levels in diabetic kidney disease rats at the 10th week after treatment. Meanwhile, the level of TC in DH group was lower than that of the NS group at the 2nd and 6th week. However, the serum of TG was not affected significantly (Figure 3).

### 3.4. DEGs Selection and KEGG Pathway Enrichment Analysis

**Figure 4:**
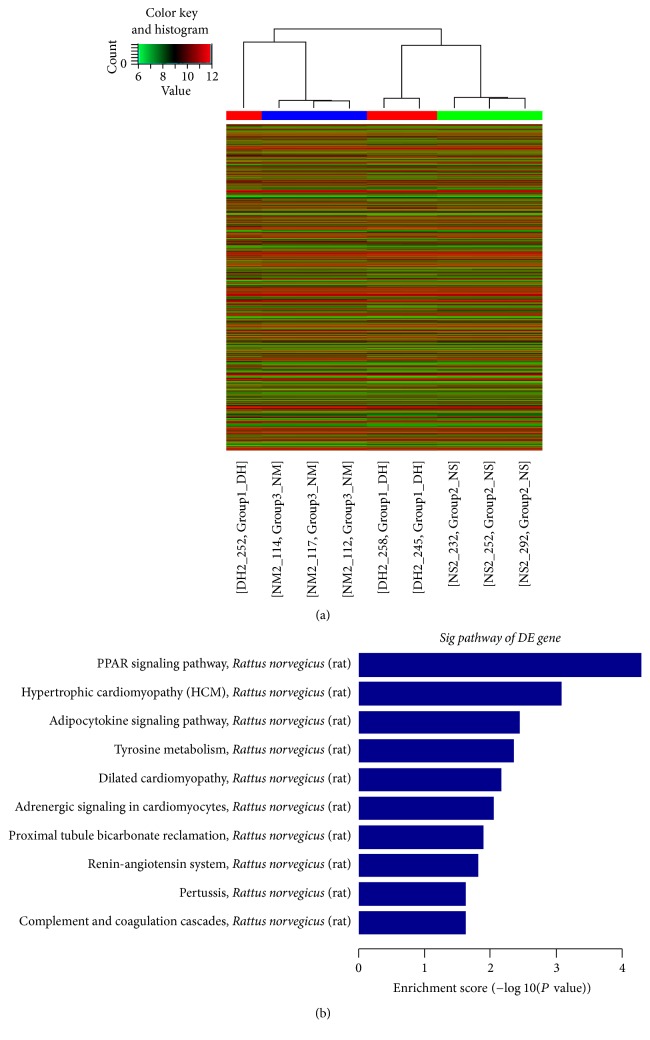
Cluster analysis and KEGG pathway enrichment analysis of differentially expressed genes. NM, NS, and DH groups, respectively, are represented by 3 samples which represent biologically independent duplicates. Red, a minimum twofold increase in expression; green, a minimum twofold reduction in expression.

The microarray assay identified a number of mRNAs that were expressed differentially between DH group and NS group. A total of 464 genes were screened out, including 185 upregulated genes and 279 downregulated genes. The hierarchical clustering of differentially expressed genes of the three groups was performed and the results were showed in Figure 4(a). The NM group and DH group were similar and they were well separated from the NS group. The KEGG pathway enrichment analysis indicated that 34 pathways were revealed to be notably affected in the DH group. The top 10 pathways upregulated by DHI are displayed in Figure 4(b). The most significant biological pathway was PPAR signaling pathway enriched with 6 genes: acyl-CoA synthetase bubblegum family member 1 (ACSBG1), adiponectin (ADIPOQ), fatty acid binding protein 4 (FABP4), similar to fatty acid translocase/CD36 (RGD1565355), solute carrier family 27 member 5 (SLC27A5), and uncoupling protein-1 (UCP-1).

### 3.5. Effects of DHI on the Expression of PPAR*γ*, UCP-1, and p38MAPK in the Kidney

**Figure 5:**
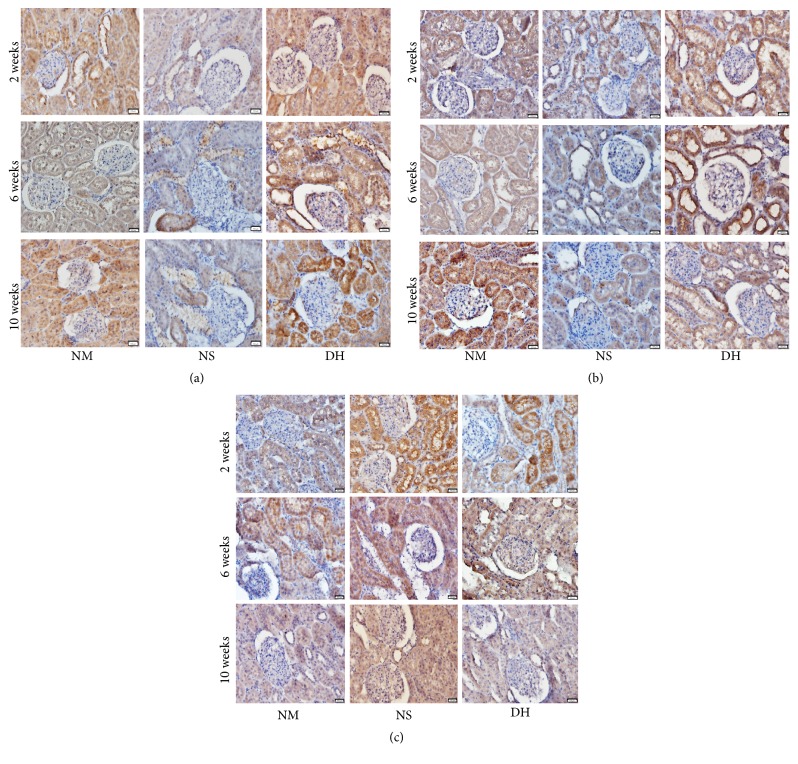
Effects of DHI on the expression of PPAR*γ*, UCP-1, and p38MAPK in the kidney. After the rats were sacrificed at the 2nd, 6th, and 10th week, the kidney 5 *μ*m cross paraffin sections were prepared and used to determine the expression of PPAR*γ* (a), UCP-1 (b), and P38MAPK (c) by immunohistochemistry (scale bar: 500 *μ*m).

To test the effect of DHI on the PPAR*γ* signaling pathway, we chose PPAR*γ* and its downstream signaling molecule UCP-1, which were both involved in PPAR*γ* signaling pathway, as well as p38MAPK, which was related to the PPAR*γ* signaling pathway, for further validation. Immunohistopathologic staining for the three proteins was performed in rat kidney tissues. As shown in Figures 5(a) and 5(b), the reduced expression levels of PPAR*γ* and UCP-1 were significantly restored after treatment with DHI. The staining of p38MAPK in NS group was much stronger than NM group (Figure 5(c)).

## 4. Discussion

The main components of DHI are tanshinone, salvia acid, salvianolic acid, safflower yellow pigment, safflower phenolic glycosides, and catechol [15]. Due to its antioxidant, antiapoptotic, anti-inflammatory, antithrombotic and antifibrinolytic effects, Danhong injection alleviated myocardial ischemia/reperfusion injury in rats [16] and minipigs [17], as well as cerebral ischemia/reperfusion injury in rats [14, 18], and exerted the protective effect in systemic acute inflammatory reaction [19], acute lung injury [20], and acute hepatic failure [21]. As for diabetic kidney disease, meta-analysis suggested that DHI can improve urinary albumin excretion rate (UAER) levels in patients [22, 23]. However, the renoprotective mechanism of DHI for diabetic kidney disease has not yet been fully defined.

Our study demonstrated that, after DHI administration, the renal function deterioration, 24-hour total urine protein excretion, and elevated serum lipid levels were reversed to some extent, and the renal pathological damage was also ameliorated. All of these data indicated that DHI has the potential renoprotective effect on STZ induced diabetic kidney disease. Moreover, the KEGG pathway enrichment analysis indicated that these effects partially depended on the activation of PPAR*γ* signaling pathway by DHI. We further validated the differential gene expression by immunohistochemistry staining. The results confirmed that the PPAR*γ* and UCP-1 were significantly decreased in kidneys of DKD rats restored after treatment with DHI. The expression of p38MAPK was activated by DHI.

Peroxisome proliferator-activated receptor gamma (PPAR*γ*) belongs to the ligand-activated type II nuclear receptor superfamily and predominantly expressed in adipose tissues [24]. In the kidney, PPAR*γ* is mainly located in the medullary collecting duct [25, 26], and glomeruli and proximal tubules were also expressed in small amounts [25]. Activated PPAR*γ* delayed the progress of DKD by improving insulin resistance [27], lowering blood pressure [28], ameliorating inflammation [29], reversing cell cycle arrest [30], increasing adiponectin [31], improving oxidative stress [32], and other mechanisms. On the other hand, the study has shown that PPAR*γ* single nucleotide polymorphism (SNP) is associated with the risk of diabetic kidney disease [33]. All of these indicated that PPAR*γ* signal pathway plays a protective role in DKD. The previous reports revealed that Danhong injection [34] and salvianolic acid B (one of the active ingredients of Danhong injection) [35] could suppress the maturation of dendritic cells through activating PPAR*γ* and thus is mediated in the therapy of metabolic and inflammatory diseases, like diabetic kidney disease. In our study, the PPAR signaling pathway was upregulated in DH group. At the same time, immunohistochemistry staining suggested that the expression of PPAR*γ* in rat renal tubules in DH group was higher than that in the NS group. It suggested that its renal protective effect was at least partially mediated by activation of PPAR*γ* signaling pathway.

In addition to the presence of glucose metabolism disorder, DKD patients also often accompany lipid metabolism disorder. An epidemiological survey showed that low HDL-C and high TG levels were independent risk factors for the development and progression of renal disease in type 2 diabetic outpatients [36]. In contrast, lowering lipid levels can protect kidney function. Yokoyama et al. found that polyunsaturated fatty acid diet can reduce the lipid deposition in the kidneys and delay the progress of DKD [37]. It is reported that DHI treatment with hyperlipidemia rats was accompanied by significantly increased lipolysis in the liver and decreased fatty acid synthesis [38]. Moreover, agonist of PPAR*γ* increased liver X receptor gene expression that mediated cholesterol efflux in glomerular mesangial cells [39] and thus reduced intracellular lipid accumulation [40]. In this study, we proved that DHI could decrease the level of serum lipids, especially cholesterol levels. So, DHI may be through PPAR*γ* signal pathway to reduce lipid levels to play its role in the protection of DKD rats.

Uncoupling protein-1 (UCP-1), which was enriched in the PPAR*γ* signal pathway predicted by the KEGG pathway enrichment analysis, is a mitochondrial inner membrane protein and stimulates thermogenesis by uncoupling oxidative phosphorylation from the respiratory chain [41]. Through binding to PPAR*γ* response elements in the UCP enhancer, referred to as UCP regulatory element 1 (URE1), PPAR*γ* positively regulated the expression of UCP in HIB-1B cells [42]. Retinoid X receptor, ligand of PPAR*γ*, was also identified as a transactivating factor of the UCP gene promoter [43]. In addition, the newly found PPAR*γ* agonist Fraglide-1 could increase expression level of the uncoupling protein- (UCP-) 1 [44]. As a downstream target gene of PPAR*γ*, UCP-1 together with PPAR*γ* played a key role in the energy balance [45, 46]. In an obesity and hyperglycemia animal model, thiazolidinedione derived partial PPAR*γ* agonist GQ-16 increased UCP-1 protein expression in interscapular brown adipose tissue (BAT) and in epididymal and inguinal white adipose tissue (WAT) to induce WAT browning and treat obesity [45]. Mu et al. reported that the browning effect of ginsenoside Rb1 on 3T3-L1 adipocytes evidenced by increased expression of UCP-1 depends on the induction of PPAR*γ* [46]. Moreover, studies indicated that UCP-1 overexpression could eliminate the production of hyperglycemia-induced reactive oxygen species (ROS) in cultured mouse glomerular mesangial cells [47], and upregulation of UCP-1 was beneficial for DKD rats [48]. In our study, we found that the protein expression levels of PPAR*γ* and UCP-1 in the rat kidney were both enhanced after DHI treatment. Therefore, we speculated that Danhong injection reduces kidney damage in diabetic rats partially through the energy metabolism regulation mediated by PPAR*γ*/UCP-1 signaling pathway.

P38 mitogen-activated protein kinase (MAPK) is a member of the MAPK family activated by environmental stresses and inflammatory cytokines. It is reported that the number of interstitial p-p38MAPK-positive cells in patients and rats with DKD reflected the severity of interstitial lesions [49] and were associated with hyperglycemia, increased HbA(1)c levels, albuminuria, and interstitial fibrosis [50]. Conversely, inhibition of the p38MAPK pathway had a beneficial effect of DKD [51]. Several studies have shown that PPAR*γ* agonists exerted therapeutic effects on various diseases by inhibiting the activity of p38MAPK [52–54]. In diabetic kidney disease, the reduced cell proliferation and fibronectin expression [55] or decreased oxidative stress and renal fibrosis [56] were associated with expression changes of PPAR*γ* and p38MAPK. In addition, Chang et al. reported that rosiglitazone could prevent AGE-induced iNOS expression by interfering with p38MAPK activity in cultured glomerular mesangial cells [57]. In a cardiac hypertrophy model in vitro, DHI downregulated the phosphorylation of p38MAPK [58]. Our results showed that DHI increased the expression of PPAR*γ* and decreased the expression of p38MAPK. Therefore, we speculated that Danhong injection delays the development of diabetic kidney disease partially through the p38MAPK/PPAR*γ* signaling pathway. This effect may be related to the inflammation regulation of P38MAPK/PPAR*γ* signaling pathway, and we need further studies to confirm it.

## 5. Conclusion

Danhong injection could delay the progress of diabetic kidney disease, and the effect might be achieved in part by activating the PPAR*γ* signaling pathway. We should explore the detailed mechanism for DHI protection against DKD in future studies.
